# Understanding barriers to women seeking and receiving help for perinatal mental health problems in UK general practice: development of a questionnaire

**DOI:** 10.1017/S1463423619000902

**Published:** 2019-12-13

**Authors:** Elizabeth Ford, Hannah Roomi, Hannah Hugh, Harm van Marwijk

**Affiliations:** Department of Primary Care and Public Health, Brighton and Sussex Medical School, Brighton, UK

**Keywords:** barriers, general practice, help-seeking, perinatal mental health, postnatal depression, questionnaire

## Abstract

**Aim:**

To develop a questionnaire to measure quantitatively barriers and facilitators to women’s disclosure of perinatal mental health problems in UK primary care. To pilot and evaluate the questionnaire for content validity and internal consistency.

**Background:**

Around 15% of women develop a mental illness in the perinatal period, such as depression, anxiety or post-traumatic stress disorder. In the United Kingdom, 90% of these women will be cared for in primary care, yet currently in as many as 50% of cases, no discussion of this issue takes place. One reason for this is that women experience barriers to disclosing symptoms of perinatal mental illness in primary care. These have previously been explored qualitatively, but no tool currently exists with which to measure these barriers quantitatively.

**Methods:**

Questionnaire items, drawn from qualitative literature and accounts of women’s experiences, were identified, refined iteratively and arranged in themes. The questionnaire was piloted using cognitive debriefing interviews to establish content validity. Women completed a refined version online. Responses were analysed using descriptive statistics. Internal consistency of subscales was calculated using Cronbach’s alpha.

**Findings:**

Cognitive debriefing interviews with five women showed the majority of questionnaire items were relevant, appropriate and easy to understand. The final questionnaire was completed by 71 women, and the majority of subscales had good internal consistency. The barrier scoring most highly was fear and stigma, followed by willingness to seek help and logistics of attending an appointment. Family/partner support and general practitioners’ (GPs) reaction were the lowest scoring barriers. Factors facilitating disclosure were GPs being empathetic and non-judgemental and listening during discussions. In the future, this questionnaire can be used to examine which barriers are most important for particular groups of women. This may enable the development of strategies to improve acknowledgement and discussion, and prevent under-recognition and under-treatment, of perinatal mental health problems in primary care.

## Introduction

Around 10–15% of women develop a mental illness in the perinatal period, which includes pregnancy and the first year after giving birth (National Institute for Health and Care Excellence, 2014). In the United Kingdom (UK), 90% of these women will be cared for in primary care, by different professionals such as midwives, nurses and general practitioners (GPs) (England, 2014). Examples of perinatal mental illnesses include antenatal and postnatal depression, anxiety, post-traumatic stress disorder (PTSD) and postpartum psychosis (Oates, 2015). Having a baby is a major and possibly stressful transition for most women, so determining the extent to which feelings of ‘distress’ are normal or could be part of a mental ‘disorder’ is a complex task for primary care clinicians.

Timely identification and treatment for perinatal mental illness is a strategic priority for health policy: mental health problems are estimated to cost UK society £8.1 billion per annual cohort of births, mostly due to the increased risk of psychological and developmental disturbances in children (Bauer *et al.*, 2014). Almost three quarters of this estimated cost relate to the adverse impacts of maternal mental illness on the child and his or her siblings, which may manifest as special educational needs, child depression, anxiety and conduct problems. As with general depression, perinatal mental illness not only affects the mother’s mental health but also her general well-being due to symptoms that range from anxiety, insomnia and irritability to loss of self, guilt and shame (Beck and Indman, 2005). In turn, these symptoms undermine the mother’s confidence, impair her social functioning and reduce her quality of life. Perinatal mental health disorders have been identified as major disruptive factors to the early mother–baby relationship (Tsivos *et al.*, 2011). In addition, mental illness is one of the leading causes of death for perinatal women, with 9% of maternal mortality caused by mental health disorders (Knight *et al.*, 2014; Knight *et al.*, 2018). Between 2009 and 2014, 111 maternal deaths in the UK were caused by suicide during pregnancy, or up to a year after childbirth (Knight *et al.*, 2014). Other leading causes of UK maternal deaths such as hypertension are at their lowest ever rate, with less than one death per one million women giving birth, due to successful evidence-based guidelines that have substantially improved patient care. However, the rate of maternal deaths from mental illness has remained largely the same since 2003 (Knight *et al.*, 2014), an issue that needs to be addressed urgently if the government’s ambition to halve maternal deaths by 2030 is to be met (Hunt, 2015).

The wider impacts of perinatal mental health highlight the implications of inadequate assessment, diagnosis and management of perinatal mental health problems in the UK and beyond and raise the question of who is responsible for postnatal care in the widest sense. In order to reduce the long-term complications of untreated mental illnesses, it is important that common perinatal mental health problems be acknowledged early, diagnosed, if needed, as serious issues, preferably in accessible primary care settings and that appropriate support or treatment is initiated without delay (Poobalan *et al.*, 2007). According to NICE clinical guidelines on Antenatal and Postnatal Mental Health, pregnant women should be asked specific depression identification questions at their first contact with primary care or at their booking visit (National Institute for Health and Care Excellence, 2014). However, only 31% of respondents in a survey reported being asked by a GP about their mental well-being and whether or not they required any additional support (Royal College of Obstetricians and Gynaecologists, 2017). As a consequence, there appears to be substantial under-recognition of postnatal depression and other perinatal disorders in general practice (Gavin *et al.*, 2015; Ford *et al.*, 2016), and it is estimated that 50% of cases of postnatal depression currently go undiagnosed (Hewitt and Gilbody, 2009). This is likely to be due to two factors. Firstly, women may find it difficult to share their experience of symptoms with their GP or other primary care clinicians and thus not ask for help. Secondly, when women do share their symptoms, GPs or other primary care staff may not identify this issue as a task within their remit, may not follow-up or make a formal diagnosis. This may be due to the fragmented nature of current postnatal care where several professionals are often involved, and where the engagement of the midwife, who might be the most accessible person to discuss ‘postpartum blues’ with, stops after the birth.

In this study, we aim to focus on the first factor, lack of sharing of perinatal mental health symptoms by women with GPs. This topic has been researched extensively using qualitative approaches. Several systematic reviews of qualitative literature on this topic have identified potential barriers and facilitators to seeking help for perinatal mental health problems (Dennis and Chung-Lee, 2006; Megnin-Viggars *et al.*, 2015; Button *et al.*, 2017). These include a reluctance to acknowledge symptoms and lack of support for mothers from partners, family members and healthcare professionals (HCPs); a lack of ability for women to talk about their feelings openly due to perceived social pressures and stigma, resulting in feelings of shame and fear of losing custody of their child; and a lack of knowledge of perinatal mental health problems among mothers, which hindered their ability to recognise and seek help for their symptoms. Valued aspects of support, such as therapeutic relationships, information and validation of difficult situations and symptoms, were key facilitators to help-seeking behaviours. These barriers and facilitators appeared to be universal across a range of countries, including mothers from different ethnicities in the UK, Canada, Australia and Japan (Hadfield and Wittkowski, 2017).

This qualitative research highlights the likely facilitators and barriers to help-seeking behaviours of women with perinatal mental health problems, but does not tell us which are the most important, and if different key barriers are more important for different groups of women, for example from different educational or cultural backgrounds. The aim of our study was therefore to develop a questionnaire to measure quantitatively previously recognised barriers and facilitators to disclosure of perinatal mental health problems in primary care settings. We focussed on appointments with GPs, because in the UK NICE guideline for antenatal and postnatal mental health, all other HCPs are advised to direct the woman to her GP if she screens positive for any mental health problem (National Institute for Health and Care Excellence, 2014). We aimed to pilot the questionnaire we created, using cognitive debriefing interviews to assess content and face validity, and to collect preliminary data to assess internal consistency and establish initial results about the respective importance of barriers women face when seeking help in general practice for perinatal mental health problems.

## Methods

### Ethical review

Both study 1 and study 2 were reviewed and received favourable ethical opinion from Brighton and Sussex Medical School Research Governance and Ethics Committee (Ref for Study 1: 16/040/FOR; Ref for Study 2: ER/BSMS2730/1).

### Questionnaire development

An initial list of items was generated for the questionnaire through analysis of theories of postnatal depression (Beck, 1993) and qualitative literature available from meta-syntheses, as well as reading online blogs, birth stories and social media forums (e.g., Facebook groups, Twitter and parenting forums such as Mumsnet) of women’s experiences in seeking help for perinatal mental health problems. Items were initially divided into barriers and facilitators and mapped onto themes from the qualitative literature. Examples of barriers included lack of knowledge about perinatal mental health problems, lack of language support services and fears about transmission of medication to breast milk. Examples of facilitators included open discussions about perinatal mental health problems to reduce stigma, and continuity of care from a single known person.

**Figure 1.**
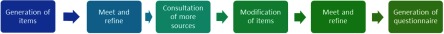
Stages of iterative refinement for questionnaire development

Expert opinion was obtained regarding the items generated to ascertain the relevance of each statement. Items were then organised into three sections: making a healthcare appointment, during the healthcare appointment and experience of support and treatment. A further subsection asked women to rate how much a range of factors influenced their access to support for perinatal mental health problems. Further sources were consulted in order to generate more items within the sections that had been outlined. Following a second expert consultation, items were refined to ensure that items were clear, concise and appropriate for response using a Likert scale, for example, double negatives and leading questions were avoided (see Figure 1). Responses to each section could be summed to produce a score representing the level of barriers.

The questionnaire was then compiled with the finalised list of items. A five-answer balanced Likert scale was used for each item consisting of strongly disagree, disagree, neutral, agree and strongly agree. Women for whom questions were not relevant could check ‘not applicable’ and this response was treated in the analysis as ‘missing’. The list of factors influencing access to support was responded to using a three-point scale (not at all; a little; a lot).

In addition, questions were included to ask women if they had made and attended a GP appointment, asked to rate the appointment and the subsequent follow-up support and to give information about the types of treatment they had been offered and had received.

### Study 1: cognitive debriefing interviews

Cognitive debriefing interviews were conducted to establish face validity and content validity of the questionnaire, as well as overall coherence, clarity and intelligibility of the questionnaire (Collins, 2003). Cognitive debriefing provides information regarding how participants go about answering survey questions while also highlighting the factors that influence the responses given. Cognitive debriefing, therefore, allows the researcher to determine whether the questions can be consistently understood or responded to by the participant in the way that was intended by the researcher, and whether the participant sees their value when measuring the construct under study.

#### Sample

Women were recruited using a convenience sampling technique. Women were eligible if they had given birth in the last three years. They were eligible to take part, whether or not they had experienced anxiety or depression and whether or not they had seen their GP.

#### Procedure

After giving informed consent, participants were interviewed in person or over the phone. Participants were given the draft questionnaire on paper and asked to complete the questionnaire verbally. While reading the questions out and giving their answers, they were asked to verbalise their overall emotional reactions to the questions, what they understood the questions were asking and whether they were clear and easily understandable. If they disliked a particular question, they were asked to suggest how it could be changed. Participants were also asked to give feedback on the usability and length of the questionnaire, the title and instructions, and the layout of items.

#### Analysis

A mixed-methods framework analysis was conducted. Comments from the audio-recordings were transcribed into a spreadsheet demonstrating each participant’s response to each theme and individual questions and stating whether the participant deemed each questionnaire item be kept, changed or removed. Discrepancies between participants’ views were addressed after discussion and consensus between two researchers (EF and HR). A table was then generated containing each questionnaire item and the outcome of the interviews. The questionnaire was refined and amended as a result of the responses; items were removed if any of the participants considered them misleading, confusing or irrelevant.

### Study 2: online survey

The amended questionnaire was taken forward to an online survey.

#### Sample

Women were eligible for inclusion in the study if they met the following criteria: female; >18 years of age; had a baby in the past two years; any symptoms of distress or feeling unable to cope whether or not help was sought. All responses were anonymous, and each participant was identified with a study number. A £50 prize draw was offered to participants as an incentive for participation.

#### Materials

The amended questionnaire was administered, alongside a demographics questionnaire (covering occupation, relationship status, living arrangements, ethnicity and age), a birth experience questionnaire (e.g., location, type and experience of birth), and brief screening tools for depression (Whooley questions), anxiety (Generalised Anxiety Disorder Scale (2-item) [GAD-2]) and post-traumatic stress disorder (The Primary Care PTSD Screen for DSM 5 [PC-PTSD-5]) (National Collaborating Centre for Mental Health, 2011; Prins *et al.*, 2016).

#### Online platform and dissemination

Qualtrics, an online survey software, was used to create an active online version of the questionnaire. The questionnaire was then piloted online with three volunteers who met the study criteria. Based on feedback received, some questions were removed due to ambiguity. The first page of the questionnaire gave full study information and participants were invited to click a button indicating their consent to participate. All partially completed questionnaires were discarded from the analysis as this was taken as a withdrawal of the participant’s consent.

The questionnaire was advertised on parenting websites in the UK such as Mumsnet and social media platforms, including Twitter and Facebook, through mums’ groups and perinatal support groups. Permission to advertise the questionnaire on any forum was gained from the administrators of the group, prior to publicising the study.

#### Analysis

Data were downloaded from Qualtrics and analysed using IBM SPSS Statistics 23, and descriptive statistics were used. Items within the questionnaire were grouped using an iterative process of refinement, to produce subscales according to themes from qualitative literature. Descriptive statistics were used to assess average responses within these themes. Cronbach’s alpha was calculated for the subscales within the questionnaire to assess internal consistency.

## Results

### Study 1

Five female participants were included in the cognitive debriefing interviews. These women had between one and three children, were mainly professional women in their 30s and two of the five had experienced a perinatal mental illness (no further demographics are given to protect participant anonymity).

The overall response to the questionnaire was generally positive. Key findings from the cognitive interviews included the need for a ‘neutral’ response option, confusion regarding who is implied by the term ‘healthcare professionals’ and the need for consistency with the use of the term ‘GP’ rather than interchanging the terms ‘GP’ and ‘doctor’.

Another key finding was that women had different experiences of perinatal mental health symptoms depending on whether it was their first pregnancy or not. For this reason, participants suggested the addition of a question at the start of the questionnaire that allows women to indicate after which birth they experienced symptoms.

Four out of the five women agreed that omission of the term ‘postnatal depression’ from the title allowed the questionnaire to be more inclusive of the women that may not relate to a formal diagnosis of perinatal mental health problems.

Items that were deemed clear, relevant and useful by all participants were kept (*n* = 79). Items that were deemed relevant but either unclear or confusing were changed (*n* = 21). Items were removed if any of the five participants considered them misleading, confusing and/or irrelevant (*n* = 8). In terms of the length and usability of the questionnaire, participants stated that the questionnaire was an appropriate length to cover all the necessary points and found that the tick-box response system was straightforward and simple to complete.

The final questionnaire is included as Appendix A.

### Study 2

#### Participants

Seventy-one women fully completed the final questionnaire, and a further 35 partially completed it; these partial results were discarded. Table 1 displays the demographic characteristics of the participants in the study. Respondents were predominantly white, married or cohabiting women in their 30s who lived in England. Ninety percent of women gave birth in hospital, and 39% had a spontaneous vaginal delivery. Participants most often rated their birth experience as disappointing or negative (59%) but were generally satisfied with the care received (extremely/fairly satisfied 65%). One of the eligibility criteria was that women should have experienced postnatal mental health symptoms; thus, our sample had a high proportion who met screening criteria for one or more of depression, anxiety and PTSD (93%).

**Table 1.** Participant demographics, birth experience, and postnatal mental health symptoms for sample 2

Categories	Number of participants	Valid percentage (%)
Age	32.85 (mean)	5.69 (Standard deviation)
Number of months since last given birth	12.28 (mean)	6.84 (Standard deviation)
Number of children		
1	48	67.6
2 or more	23	32.4
Ethnicity		
White	65	91.5
Mixed/Multiple ethnic groups	5	7.0
Chinese	1	1.4
Highest level of education		
GCSE	7	9.9
A level or equivalent	17	23.9
Bachelor’s degree or above	47	66.2
Current occupation		
Student	2	2.8
Employed	47	66.2
Self-employed	7	9.9
Stay-at-home parent	12	16.9
Unemployed	1	1.4
Other	2	2.8
Current relationship status		
Single	2	2.8
In a relationship/married	69	97.2
Overall experience of birth		
Good/reasonable	29	40.8
Disappointing/negative	42	59.2
Satisfaction of care received during birth		
Extremely/fairly satisfied	46	64.8
Neither satisfied nor dissatisfied	6	8.5
Extremely/Fairly dissatisfied	19	26.8
Physical problems present since birth		
No	42	59.2
Yes	29	40.8
Number of participants meeting threshold for postnatal mental health disorder		
Any disorder	66	93.0
Depression	63	88.7
Anxiety	46	63.0
Post-traumatic stress disorder	25	34.2

#### Engagement with and evaluation of GP services for perinatal mental health problems

Although 93% (*n* = 66) of the sample reported experiencing mental health symptoms which met screening criteria, only 41 (58%) reported attending an appointment with their GP. Experience of this appointment can be seen in Table 2. In this sample, 29% rated their initial GP appointment about symptoms as poor or very poor, and 46% rated follow-up support as poor or very poor. During or following this appointment, a total of 34 of 41 women (83%) were offered antidepressants, of whom 31 received them (76%). The next most commonly offered therapy was cognitive behavioural therapy, which was offered to 12 women (29%) and received by 10 (24%). Other forms of therapy were reported by even fewer women, and four women (10%) reported being offered no treatment, with five women (12%) reporting that they received no treatment.

**Table 2.** Evaluation of GP appointments and treatment provided (*n* = 41, 58%)

	Response (valid percent)				
Question	Very good	Good	Neutral	Poor	Very poor
How would you rate your initial appointment about your symptoms?	8 (20%)	16 (39%)	5 (12%)	9 (22%)	3 (7%)
How would you rate any follow-up support you were offered after the healthcare appointment?	6 (15%)	12 (29%)	4 (10%)	12 (29%)	7 (17%)

#### Average scores on questionnaire items: which barriers score most highly?

Table 3 shows that the group of barriers with the highest mean score (3.68) were related to fear and stigma, where a high score (range 1–5) indicates greater perception of a barrier. Willingness to seek help (3.38) and logistics of getting an appointment (3.11) and attending an appointment (3.27) were also above the average score of all barriers (3.02). The groups of barriers with the lowest mean scores were related to family and partner support (2.12) and GPs’ reaction (2.41).

**Table 3.** Average score on barriers and Cronbach’s alpha (high score = worse barrier)

Barrier theme	*N* items	Mean score (range 1–5)	Standard deviation	Cronbach’s alpha
Self-recognition	6	2.79	0.48	0.66
Willingness to seek help	4	3.31	0.86	0.65
Fear and stigma	5	3.68	0.94	0.81
Family/partner support	2	2.12	0.89	0.49
Logistics of getting an appointment	12	3.11	0.66	0.77
GP reaction	17	2.41	0.77	0.91
Logistics of attending appointment	8	3.27	0.71	0.58
Barriers to therapy	12	3.10	1.02	0.86
Mean of all barriers	66	3.02	0.41	–

Cronbach’s alpha was calculated as a measure of internal consistency within the subscales and the results are shown in Table 3. Subscales showing acceptable to good internal consistency [at least 0.70 (Tavakol and Dennick, 2011)] were fear and stigma, logistics of getting an appointment, GP reaction and barriers to therapy. Given the low alphas for self-recognition, willingness to seek help and logistics of attending an appointment, we evaluated whether alpha could be increased by removing one or more item which was proving inconsistent with the others. As a result of this process, we removed one item from self-recognition ‘I thought I was struggling’ as this would increase alpha to 0.72. No items were removed from willingness to seek help as these all correlated highly with the total scale. We removed one item from logistics of attending an appointment ‘a translator was available’ as this would increase alpha to 0.67. The low alpha for family/partner support was assumed to be due to the short scale (Tavakol and Dennick, 2011).

#### Factors influencing women’s perception of access to support for perinatal mental health problems

The four factors with the highest mean scores (range 1–3) were ‘HCPs being empathetic and non-judgemental’ (2.89), ‘Having my voice heard in discussions and decisions about treatment’ (2.83), ‘Partners that encourage women to seek help’ (2.80) and ‘Opportunity to build trust and respect with HCPs’ (2.77). The four factors with the lowest mean scores were ‘Social media e.g. Facebook, Twitter, Instagram’ (1.92), ‘Internet searches about symptoms’ (2.01), ‘Internet forums and communities/ blogs’ (2.14) and ‘Childcare when attending my appointment’ (2.46). These are shown in Table 4.

**Table 4.** Average score for influencing factors

Influencing factor	Mean score (range 1–3)	Standard deviation
Mean of all influencing factors	2.54	0.33
Digital health help-seeking		
Internet searches about symptoms	2.01	0.69
Social media, for example, Facebook, Twitter, Instagram	1.92	0.73
Internet forums and communities/blogs	2.14	0.66
Interpersonal factors with healthcare professionals		
Having my voice heard in discussions and decisions about treatment	2.83	0.45
Honest discussions with GP about medications to get full information	2.74	0.53
Opportunity to build trust and respect with healthcare professionals	2.77	0.48
Healthcare professionals being empathetic and non-judgemental	2.89	0.32
Continuity of care from a single known person	2.72	0.54
Close relationships with health professionals	2.55	0.65
Support from friends and family		
Friends who encourage women to seek help	2.63	0.59
Partners who encourage women to seek help	2.80	0.50
Childcare when attending my appointment	2.46	0.74
Knowledge		
Previous knowledge about postnatal mental health problems	2.52	0.58

## Discussion

To the authors’ knowledge, this is the first questionnaire to examine quantitatively women’s experiences, barriers and facilitators for seeking help for postnatal mental health problems, in primary care. We developed the questionnaire and piloted it in a two-stage process. First, we undertook in-depth cognitive debriefing interviews with 5 women, and then 71 women completed the final questionnaire to check for average scores and internal consistency of the scales. The most important barriers to help-seeking were fear and stigma, self-recognition, willingness to seek help and the logistics of attending an appointment. These all relate to challenges around choosing to seek help.

We developed our questionnaire using an iterative process of item generation and refinement and consulted with end-users at several stages of the process. Previous questionnaires have studied similar phenomena, the best known is Meadows *et al.*’s Perceived Need for Care Questionnaire (2000), which, in one subscale, examines the barriers to perceived needs being met, similar to our ‘before the appointment’ section. These include self-reliance, pessimism about treatment efficacy, lack of knowledge and social stigma. An interesting future study would be to also administer both our questionnaire and the Meadows’ onto another sample of similar participants to establish criterion validity. In this study, we evaluated face and content validity using a cognitive debriefing method which helped us to refine our questionnaire and check that each question was relevant and purposeful. We also found reasonable internal consistency within the majority of sub-scales, although the low alpha coefficients for three of the subscales led to two items being removed to improve consistency.

Ninety-three percent of the participants screened positive for at least one of the mental health conditions (depression, anxiety or PTSD), as would be expected from the inclusion criteria. Nevertheless, 42% of participants did not go to see their GP, suggesting that the primary barriers women experience may be those which prevent them from going to the GP in the first place. These results are supported by the similar findings by Dennis and Chung-Lee (2006) who identified that mothers opening up and talking to someone about their feelings were major barriers to help-seeking behaviours. They went on to suggest that when women are unwilling or unable to attend clinics that services should be provided to their home, for example, through telephone consultations (Dennis and Chung-Lee, 2006). Awareness that these services exist could help to increase the disclosure of perinatal mental health symptoms. Furthermore, they identified that on average, mothers had a preference for psychological treatments instead of medications (Dennis and Chung-Lee, 2006). In areas where psychological treatments are believed to be difficult to access, this may act as a barrier to help-seeking behaviours. Of additional interest is the fact that the logistics of attending an appointment scored highly as a barrier. It should be noted when planning primary care services that women who are caring for small children with little support, possibly also trying to manage school or nursery runs for older children, and possibly going to work, may find attending a healthcare appointment difficult, or when there, may find it hard to disclose details of their current mental health difficulties.

Interestingly, despite nearly a third of participants saying that their initial GP appointment about perinatal mental health problems was poor or very poor, and nearly half saying that follow-up support was poor or very poor, one of the lowest scoring barriers was related to GP’s reactions. This apparent discrepancy in responses is hard to explain. As not every participant saw their GP, there were fewer responses to the questions based on the GP’s reactions, logistics of attending the appointment and the barriers to therapy, meaning responses to these items may be less representative than the sample as a whole. Dolman *et al.* (2013), Megnin-Viggars *et al.* (2015) and Button *et al.* (2017) identified the GP’s reactions in consultations as being a significant barrier to disclosure of symptoms and access to support for perinatal mental health symptoms. These findings were partially reflected in our study, given that the most important influencing factors for symptom disclosure were endorsed as HCPs listening, being open and honest, empathetic and enabling a relationship of trust and respect.

This study suggests the most influential factor, facilitating access to disclosure of symptoms, is therefore related to elements of interpersonal relationships with HCPs, family and friends and the woman’s involvement in making decisions about her management. Mirroring these findings, Hadfield and Wittkowski (2017) found that therapeutic relationships with HCPs were key factors in enabling women to disclose their symptoms of perinatal mental health problems. Moreover, caring relationships and mothers being enabled to discuss their feelings with a HCP in an unrushed, conversational manner instead of a questionnaire format are associated with increased acceptability of screening experiences and access to support (Ayers, 2004; Armstrong and Small, 2010).

### Strengths and limitations

Our small sample, and the novelty of the concept being measured, limited our ability to test various psychometric properties of the questionnaire. One such property is the underlying factor structure of the questionnaire. Our small sample meant conducting a principal component analysis (PCA) was not possible, although given no widely accepted theoretical structure of help-seeking barriers has been proposed in the literature (Rickwood and Thomas, 2012), we would have had no way of validating the results of a PCA. However, future work, where the questionnaire is used with a larger sample, should assess the structure of the subscales statistically, and the questionnaire may need further refinement.

Additionally, we did not measure criterion validity, given most existing health help-seeking questionnaires do not focus on postnatal women’s specific circumstances and challenges. Thus, we chose to focus on consultation with end-users as our primary method to establish content validity of the questions. The women we consulted in study 1 were largely very positive about the questionnaire and agreed that although it was long, the majority of the questions were performing a useful purpose and covered aspects of help-seeking which were meaningful to them. Following feedback during study 1, we changed broader terms such as ‘healthcare professionals’ to ‘GP’ for simplicity and clarity. This decision means the questionnaire is currently focussed on appointments with the GP. In future studies the wording of questionnaire items could be changed to focus specifically on other professional groups such as health visitors or could be changed back to a broader term to encompass all clinicians who care for women during the postnatal period.

We evaluated internal consistency of the subscales and found this was largely adequate to good, although results for some of the scales could be improved by removal of some of the items. Some scales were too short to get meaningful internal consistency measurements (e.g., family and partner support).

The small sample also meant that we were unable to identify differences on responses to barriers between women with different demographic characteristics, birth experiences or mental health symptoms. The participants lacked diversity in their demographic characteristics, especially regarding ethnicity, country of residence, relationship status and living situations. This meant that we were not able to test for differences in experiences of barriers between different groups. In addition, our recruitment strategy via the internet may have meant that not all women meeting the inclusion criteria would have had access to the websites or social media platforms (Facebook, Twitter, etc.); thus, our sample may not be representative of all the types of women we would wish to reach.

### Future directions

In future research, when using this questionnaire with larger samples, researchers will be able to test a range of hypotheses that women with different demographic characteristics, birth experiences and symptoms experience differently the barriers to help-seeking for perinatal mental health. In future research, we might expect to find that women with anxiety disorders may experience more barriers due to fear and stigma than other mental health conditions (Woolhouse *et al.*, 2009). It has also been found previously that depression and its risk factors are associated with reduced rates of self-recognition of mental health symptoms, and that mothers’ previous experience of treatment, either personally or through friends and family, influences their decisions regarding their future management (Sealy *et al.*, 2009; Bowen *et al.*, 2012; Buist *et al.*, 2015). Additionally, previous research has found a disparity in the accessibility or availability of mental health services between different educational, cultural and socioeconomic settings (Saxena *et al.*, 2007). Dennis and Chung Lee (2006) emphasised the importance of understanding the influence of cultural barriers to help-seeking behaviours. For example, the term ‘postnatal depression’ may not be culturally acceptable to many mothers, and alternative approaches to identification and management may be required, including the use of terms such as ‘tension’. Women from migrant backgrounds, particularly during the perinatal period, are more exposed to barriers when identifying and seeking help for their mental health than those from non-migrant backgrounds (Bakshi *et al.*, 1999; Buist *et al.*, 2015). Echoing this, barriers to identification were among the highest scoring barriers in this study, despite the lack of demographic diversity.

An additional avenue that could be explored in the future would be primary care practitioners’ (PCPs) perspectives on barriers to disclosure for women with perinatal mental health problems and their access to treatment. A study involving midwives highlighted that they felt able to determine which patients needed extra consultation time, more efficiently, once they had been trained to screen and refer for depression during pregnancy (Brugha *et al.*, 2011). Moreover, understanding PCPs’ perspectives on the barriers they encounter when discussing perinatal mental health problems with patients is vital in order to improve service provision. A qualitative study found that many patients were quick to pick up on HCPs’ unease when discussing depression. In conjunction with patient disquiet this may elicit an overall sense of discomfort during the consultation (Rollans *et al.*, 2013a; 2013b).

## Conclusions

We present a new, evaluated questionnaire for quantitatively measuring women’s experiences and barriers to disclosing and seeking help for common perinatal mental health problems, especially developed for primary care. This questionnaire, developed from the qualitative literature, has good content validity and adequate internal consistency. While developed in the UK, this questionnaire can be used with women in any health system which has a similar structure to the UK. In the future, it can be used to examine which barriers are most important for particular groups of women. This approach has the potential to enable strategies to be developed which tackle the current problem of under-recognition and under-treatment of perinatal mental health in the UK.

## Supplementary material

For supplementary material accompanying this paper visit https://doi.org/10.1017/S1463423619000902. click here to view supplementary material
