# Supplementary material for: Understanding barriers to women seeking and receiving help for perinatal mental health problems in UK general practice: development of a questionnaire
Source: Prim Health Care Res Dev. 2019 Dec 13;20:e156. doi: 10.1017/S1463423619000902 (PMC7003527; doi:10.1017/S1463423619000902)
Supplement: Supplementary file 1 [file S1463423619000902sup001.docx]

**Understanding women’s experiences seeking help for feeling anxious, overwhelmed and/or struggling to cope after having a baby**

Q1 Some of your details:

o How old are you?

o How many months ago did you give birth?

o How many children do you have?

Q2 Please state after the birth of which child you experienced symptoms of sadness, depression, anxiety or stress e.g. "1st of 3" or "1st and 2nd of 2".

Q3 Did you make an appointment with your GP to discuss seeking help for any symptoms of sadness, depression, anxiety or stress?

(Click the appropriate answer)

o Yes

o No

| **Making an Appointment**  Q4 To what extent would you agree with the following statements: | | | | | | |
| --- | --- | --- | --- | --- | --- | --- |
|  | Strongly Disagree | Disagree | Neutral | Agree | Strongly Agree | Not applicable |
| I thought I was struggling | o | o | o | o | o | o |
| I felt that I could deal with my feelings myself | o | o | o | o | o | o |
| I thought this was a normal part of motherhood | o | o | o | o | o | o |
| I did not understand the way I was feeling | o | o | o | o | o | o |
| I did not know when to seek help for the way I was feeling | o | o | o | o | o | o |
| I did not know what postnatal distress/ sadness/ worry was | o | o | o | o | o | o |
| I was keen to get treatment as soon as possible | o | o | o | o | o | o |
| I understood the way I was feeling | o | o | o | o | o | o |
| I did not want to accept that I was not well | o | o | o | o | o | o |
| I felt guilty about what I was experiencing | o | o | o | o | o | o |
| I was too exhausted to take any positive action | o | o | o | o | o | o |
| I felt that the health of my baby was more of a priority to health professionals than mine | o | o | o | o | o | o |
| I was afraid as being labelled "mentally ill" | o | o | o | o | o | o |
| I was afraid that I would lose my baby | o | o | o | o | o | o |
| I was afraid of being labelled a "bad mother" | o | o | o | o | o | o |
| I viewed depression as a sign of weakness | o | o | o | o | o | o |
| My family discouraged me from seeking help | o | o | o | o | o | o |

| I was encouraged by my partner to tell a healthcare professional how I felt | o | o | o | o | o | o |
| --- | --- | --- | --- | --- | --- | --- |
| There was an opportunity to raise my concerns at the baby weigh-in sessions | o | o | o | o | o | o |
| None of the healthcare professionals seemed interested in my mental health | o | o | o | o | o | o |
| The appointment making process was straight forward | o | o | o | o | o | o |
| The waiting time for an appointment was short | o | o | o | o | o | o |
| A health professional asked me about my mental health | o | o | o | o | o | o |
| I did not know what kind of support would be available | o | o | o | o | o | o |
| I did not know where to go for help for the way I was feeling | o | o | o | o | o | o |
| I am familiar with seeking mental health advice | o | o | o | o | o | o |
| I did not know what I would say to my GP | o | o | o | o | o | o |
| I did not go to the GP as I thought they would not understand | o | o | o | o | o | o |
| I did not feel my GP was the best place to seek help | o | o | o | o | o | o |

Q5 If you agreed with the following statement;

'I did not feel my GP was the best place to seek help', then who did you seek help from?

**During the Appointment**

Q6 Did you attend the appointment you made with your GP? (Click the appropriate)

o Yes

o No

o Not Applicable

Q6(a) How would you rate your initial appointment about your symptoms? (Click the appropriate)

o Very Poor

o Poor

o Neutral

o Good

o Very Good

Q6(b) To what extent would you agree with the following statements: (Click the appropriate)

|  | | Strongly Disagree | | Disagree | | Neutral | | Agree | | Strongly Agree | | Not Applicable | | |
| --- | --- | --- | --- | --- | --- | --- | --- | --- | --- | --- | --- | --- | --- | --- |
| The GP understood my symptoms | | o | | o | | o | | o | | o | | o | | |
| The GP made me feel at ease | | o | | o | | o | | o | | o | | o | | |
| The GP was sympathetic | | o | | o | | o | | o | | o | | o | | |
| The GP said my symptoms were not important | | o | | o | | o | | o | | o | | o | | |
| The GP had a patronising attitude towards my symptoms | | o | | o | | o | | o | | o | | o | | |
| The GP seemed too busy to listen to my concerns | | o | | o | | o | | o | | o | | o | | |
| The GP was more interested in the needs of the baby | | o | | o | | o | | o | | o | | o | | |
| The GP did not understand what I was trying to say | | o | | o | | o | | o | | o | | o | | |
| The GP was knowledgeable about mental health problems | | o | | o | | o | | o | | o | | o | | |
| The GP assured me that my confidentiality would be maintained | | o | | o | | o | | o | | o | | o | | |
| The GP avoided medical jargon | | o | | o | | o | | o | | o | | o | | |
| The GP did not rush the appointment | | o | | o | | o | | o | | o | | o | | |
| The GP interrupted me before I had finished explaining the way I was feeling | | o | | o | | o | | o | | o | | o | | |
| The GP was only concerned with my physical health | | o | | o | | o | | o | | o | | o | | |
| I was able to see a GP I already knew well | | o | | o | | o | | o | | o | | o | | |
| The healthcare professional told me it was "normal" to feel as low as I did | | o | | o | | o | | o | | o | | o | | |
| I felt like the GP was just looking for a quick solution | | o | | o | | o | | o | | o | | o | | |
| I could arrange suitable childcare while attending my appointment | | o | | o | | o | | o | | o | | o | | |
| A translator was available | | o | | o | | o | | o | | o | | o | | |
| The environment where I had the appointment was comfortable | | o | | o | | o | | o | | o | | o | | |
| The appointment was on time | | o | | o | | o | | o | | o | | o | | |
| I saw the gender of healthcare professional that I wanted to | | o | | o | | o | | o | | o | | o | | |
| I felt comfortable being open with the healthcare professional | | o | | o | | o | | o | | o | | o | | |
| I struggled to know what the right words were to express my symptoms | | o | | o | | o | | o | | o | | o |  |  |
| The support my partner offered for attending the appointment was just right for me | | o | | o | | o | | o | | o | | o |  |  |

**After the Appointment**

Q6(c)

How would you rate any follow up support you were offered after the healthcare appointment? (Click the appropriate)

o Very Poor

o Poor

o Neutral

o Good

o Very Good

Q6(d) What treatments/ therapies were offered to you by your GP? (Please click all the appropriate options)

▢None

▢Antidepressants

▢Guided self-help

▢Cognitive behavioural therapy (CBT)

▢Interpersonal therapy

▢Group therapy

▢Computer-aided CBT

▢Other

Q7 To what extent would you agree with the following statements:

(Click the appropriate)

|  | Strongly Disagree | Disagree | Neutral | Agree | Strongly Agree | Not Applicable |
| --- | --- | --- | --- | --- | --- | --- |
| The waiting list for therapy was too long | o | o | o | o | o | o |
| There was a lack of talking therapies available through my GP | o | o | o | o | o | o |
| I was not offered any childcare support while receiving treatment | o | o | o | o | o | o |
| The support was not culturally relevant | o | o | o | o | o | o |
| My cultural preferences were taken into consideration | o | o | o | o | o | o |
| The care-plan I received was personalised | o | o | o | o | o | o |
| I was involved in making decisions about my care | o | o | o | o | o | o |
| I was not offered any support outside my GP | o | o | o | o | o | o |
| I have always been able to talk to the same healthcare professional about my issues | o | o | o | o | o | o |
| Antidepressants have helped manage my symptoms | o | o | o | o | o | o |
| Cognitive behavioural therapy changed my life for the better | o | o | o | o | o | o |
| I was supported in developing my own coping strategies | o | o | o | o | o | o |

Q6(e) Which treatments/ therapies did you actually receive? (Please click all the appropriate options)

▢None

▢Antidepressants

▢Guided self-help

▢Cognitive behavioural therapy

▢Interpersonal therapy

▢Group therapy

▢Computer-aided therapy

▢Other

Q8 If you sought help through alternative therapies, which therapies did you use? (Click all appropriate options)

▢Meditation

▢Yoga

▢St John's Wort

▢Community Faith Groups

▢Other

Q8(a) If you selected other for the previous question, which therapy did you use?

Q9

To what extent do you agree that these factors make it easier to seek help? (Click the appropriate)

|  | Not At All | A Little | A Lot |
| --- | --- | --- | --- |
| Previous knowledge about post-natal mental health problems | o | o | o |
| Childcare when attending my appointment | o | o | o |
| Partners that encourage women to seek help | o | o | o |
| Friends that encourage women to seek help | o | o | o |
| Close relationships with health professionals | o | o | o |
| Continuity of care from a single known person | o | o | o |
| Health professionals being empathetic and non-judgemental | o | o | o |
| Opportunity to build trust and respect with healthcare professionals | o | o | o |
| Honest discussions with GP about medications to get full information | o | o | o |
| Having my voice heard in discussions and decisions about treatment | o | o | o |
| Internet forums and communities/ blogs | o | o | o |
| Social media e.g. Facebook, Twitter, Instagram | o | o | o |
| Internet searches about symptoms | o | o | o |

Q10 Which websites did you use/ find helpful?
